# A longitudinal study defined circulating microRNAs as reliable biomarkers for disease prognosis and progression in ALS human patients

**DOI:** 10.1038/s41420-020-00397-6

**Published:** 2021-01-11

**Authors:** Gabriella Dobrowolny, Julie Martone, Elisa Lepore, Irene Casola, Antonio Petrucci, Maurizio Inghilleri, Mariangela Morlando, Alessio Colantoni, Bianca Maria Scicchitano, Andrea Calvo, Giulia Bisogni, Adriano Chiò, Mario Sabatelli, Irene Bozzoni, Antonio Musarò

**Affiliations:** 1 DAHFMO-Unit of Histology and Medical Embryology, Sapienza University of Rome, Laboratory Affiliated to Istituto Pasteur Italia-Fondazione Cenci Bolognetti, Via A. Scarpa 14, 00161 Rome, Italy; 2 Institute of Molecular Biology and Pathology, National Research Council, Sapienza University of Rome, 00185 Rome, Italy; 3 Centre for Neuromuscular and Neurological Rare Diseases, San Camillo Forlanini Hospital, 00152 Rome, Italy; 4 Rare Neuromuscular Diseases Centre, Department of Human Neurosciences, Sapienza University of Rome, 00185 Rome, Italy; 5 Department of Pharmaceutical Sciences, “Department of Excellence 2018-2022”, University of Perugia, Perugia, Italy; 6 Center for Life Nano Science@Sapienza, Istituto Italiano di Tecnologia, Rome, Italy; 7 Sezione di Istologia ed Embriologia, Dipartimento di Scienze della Vita e Sanità Pubblica, Fondazione Policlinico Universitario A. Gemelli IRCCS, 00168 Roma, Italy; 8 Department of Neuroscience, Molinette Hospital, Turin, Italy; 9 Department of Geriatrics, Neuroscience and Orthopedics, Institute of Neurology, Catholic University, Rome, Italy; 10 Department of Biology and Biotechnology “Charles Darwin”, Sapienza University of Rome, 00185 Rome, Italy

**Keywords:** Prognostic markers, Amyotrophic lateral sclerosis

## Abstract

Amyotrophic lateral sclerosis (ALS) is a fatal neurodegenerative disease associated with motor neuron degeneration, muscle atrophy and paralysis. To date, multiple panels of biomarkers have been described in ALS patients and murine models. Nevertheless, none of them has sufficient specificity and thus the molecular signature for ALS prognosis and progression remains to be elucidated. Here we overcome this limitation through a longitudinal study, analyzing serum levels of circulating miRNAs, stable molecules that are recently used as promising biomarkers for many types of human disorders, in ALS patients during the progression of the pathology. We performed next-generation sequencing (NGS) analysis and absolute RT quantification of serum samples of ALS patients and healthy controls. The expression levels of five selected miRNAs were quantitatively analyzed during disease progression in each patient and we demonstrated that high levels of miR-206, miR-133a and miR-151a-5p can predict a slower clinical decline of patient functionality. In particular, we found that miR-206 and miR-151a-5p serum levels were significantly up-regulated at the mild stage of ALS pathology, to decrease in the following moderate and severe stages, whereas the expression levels of miR-133a and miR-199a-5p remained low throughout the course of the disease, showing a diagnostic significance in moderate and severe stages for miR-133a and in mild and terminal ones for miR-199a-5p. Moreover, we found that miR-423–3p and 151a-5p were significantly downregulated respectively in mild and terminal stages of the disease. These data suggest that these miRNAs represent potential prognostic markers for ALS disease.

## Introduction

Amyotrophic lateral sclerosis (ALS) is a fatal neurodegenerative disease associated with motor neurons degeneration, muscle atrophy and paralysis^1^. In addition, energy balance is severely compromised in ALS patients, owing to higher consumption than intake with increased resting energy use, along with abnormal lipid metabolism^2–6^.

Diagnosis of ALS relies on clinical, electrophysiological or neuropathological examination and is based on the exclusion of alternative related pathologies^7,8^.

ALS can proceed with different aggressiveness and velocity; nevertheless, the characterization of specific molecular mechanisms and potential molecular biomarkers of the progression of the disease remain to be fully elucidated. Thus, the definition of biomarkers of ALS that can be included in the diagnosis and prognosis of the disease, will help to better restrict the phenotypes of ALS, and where appropriate, to enroll the patients with a specific molecular signature in clinical trials.

MicroRNAs (miRNAs) are evolutionary conserved non coding RNA molecules with post-transcriptional regulatory role and are frequently dysregulated in human disease^9–11^. Moreover, tissue-specific miRNAs, normally restricted in the tissue of expression, can be released in the blood, under pathological conditions. Thus, there is an increase interest to define their role in the pathogenesis of several degenerative diseases, as minimally invasive and low- cost disease biomarkers^12^.

Recent studies report the identification of potential circulating miRNAs, namely miR-4649–5p, miR-424, miR-133a, and miR-206, as putative serum biomarkers for ALS diagnosis^13–15^.

To add further insights into the potential molecular biomarkers of ALS and to define potential miRNA signature for ALS progression, we performed next-generation sequencing (NGS) using serum from both ALS patients and healthy controls and identified a general downregulation of miRNAs expression in ALS patients respect to controls.

Here we analyzed circulating levels of five specific miRNAs (miR-206, miR-133a, miR-199a-5p, miR-151a-5p, and miR-423–3p) in sera, periodically collected every 3 months, from ALS patients. Through a longitudinal analysis, we demonstrated that early phases of ALS pathology are characterized by low levels of miR-199a-5p, miR-133a and mir-423–3p and by high levels of miR-151a-5p and miR-206, which also predicted a slower clinical decline of patient functionality, suggesting that these miRNAs can represent potential prognostic markers for ALS disease and valid biomarkers to stratify the severity of disease progression.

## Results

### Differential expression of serum miRNAs in ALS patients compared to healthy controls

**Fig. 1:**
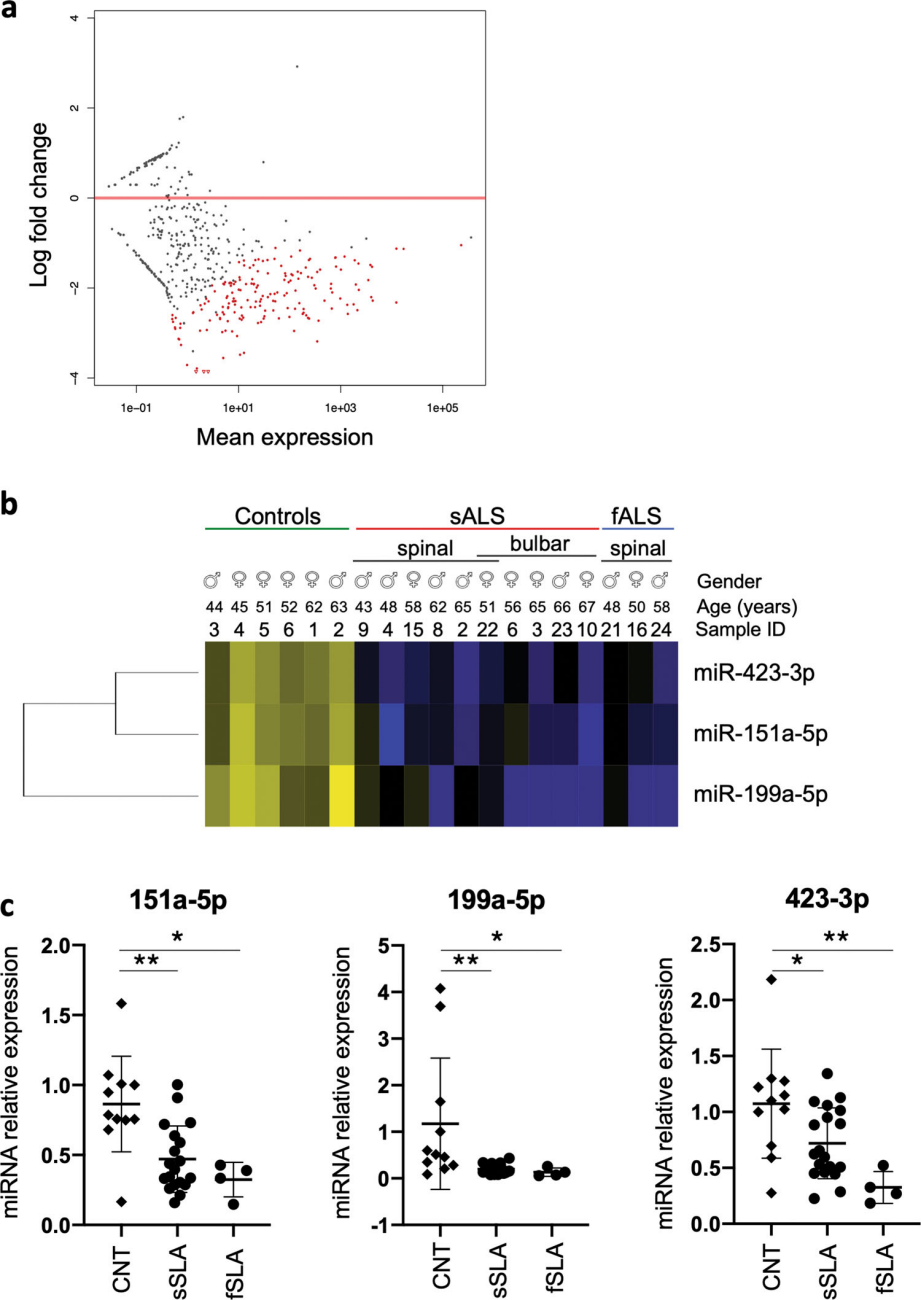
NGS analysis of miRNAs expression in healthy and ALS patients. **a** MA plot and **b** expression heatmap of miRNAs expression in healthy and ALS patient sera showing selected downregulated serum miRNAs from sALS and fALS patients compared with healthy controls (CNT). Blue represents downregulation and yellow represents upregulation. Gender, age, and samples ID are indicated. In the scatter plot, log2 fold change of miRNA expression values between healthy and ALS patient sera are plotted against the mean of DESeq2 miRNA normalized read counts. Negative fold changes reflect depletion in ALS patient sera. Dots are colored red if the adjusted *p*-value for the differential expression test is less than 0.1. **c** qPCR validation by relative quantification of miR-151a-5p, miR-199a-5p and miR-423-3p in serum samples of 19 sALS and 4 fALS patients with respect to 11 healthy controls. All data were normalized for a spiked miRNA (cel-lin-4) and expressed as fold change with respect to a healthy control set to a value of 1.

To gain insight into the circulating miRNAs expression in ALS, NGS was performed, using Small RNA sequencing technology (Illumina), on RNA isolated from serum of 13 ALS patients along with 6 matched healthy controls. The cohort analyzed was constituted of 10 sporadic (sALS, mean age 58.1 ± 8.4) and 3 familiar (fALS, mean age 54 ± 5.7) ALS patients comprising 7 males and 6 females; among them 4 were characterized by bulbar onset, 8 by spinal onset, while one by both spinal/bulbar onset (Supplementary Fig. S1a). The abundance of microRNAs analyzed was normalized towards a synthetic endogenous control (cel-lin-4) that was added to the serum samples before the RNA extraction. The sequencing revealed a clear clustering of ALS samples on one side, and of healthy controls on the other side (Supplementary Fig. S1b). Interestingly, we identified 585 miRNAs expressed in all the serum samples analyzed and we observed a significant downregulation of 152 miRNAs of them, with no miRNAs significantly upregulated (Fig. 1a).

In order to identify suitable biomarkers candidates, we selected, from NGS data, three relevant miRNAs (miR-151a-5p, miR-199a-5p, miR-423–3p) that resulted significantly downregulated in ALS patients compared to healthy controls (Fig. 1b).

The decreased expression levels of the miRNAs, identified by NGS, were validated by qPCR in sera derived from different cohort of ALS patients and healthy control subjects, namely 9 males and 10 females sALS patients (mean age 56.6 ± 10.9), 2 males and 2 females fALS patients (mean age 56 ± 9.9) and 11 matched healthy controls (6 males and 5 females, mean age 50.0 ± 10.1) (Supplementary Fig. S1,c).

The three miRNAs (miR-151a-5p, miR-199a-5p, miR-423–3p), were robustly and significantly downregulated in both sporadic and familiar ALS cases compared to healthy controls (Fig. 1c).

### MiRNAs expression levels are differentially modulated in ALS patients during disease progression

To assess the role of selected miRNAs as prognostic biomarkers, their expression levels were quantified in serum samples from healthy controls (age 55.0 ± 11.2) and patients (age 69.5 ± 9.6) that were collected every 3 months for a maximum of 30 months of follow-up. We analyzed both male and female with diagnosed spinal onset ALS disease and by ALS Functional Rating Score (ALS-FRS) (Table 1).

**Table 1:** Demographic and clinical data of patients and healthy controls included in the study.

Characteristics	ALS Patients (*n* = 27)		Healty Controls (*n* = 13)	
Age (Mean ± SD, years) Gender (M/F) Age at onset (Mean ± SD, years) ALS-FRS Score at time of enrolment (Mean ± SD)	69.5 ± 9.6 17/10 60.6 ± 13.1 36.2 ± 6.4		55.0 ± 11.2 8/5 / /	
ALS-HSS N. samples time of enrolment	**LEVEL I:** 3	**LEVEL II:** 8	**LEVEL III:** 4	**LEVEL IV:** None
N. samples	13	16	20	9

Clinical course of ALS pathology is highly variable and the duration of the disease ranges from months to more than two decades after onset^16^. In order to disclose a possible difference in disease progression of patients enrolled in our study, we evaluated the decline in the clinical rate of patients enrolled in the study.

**Fig. 2:**
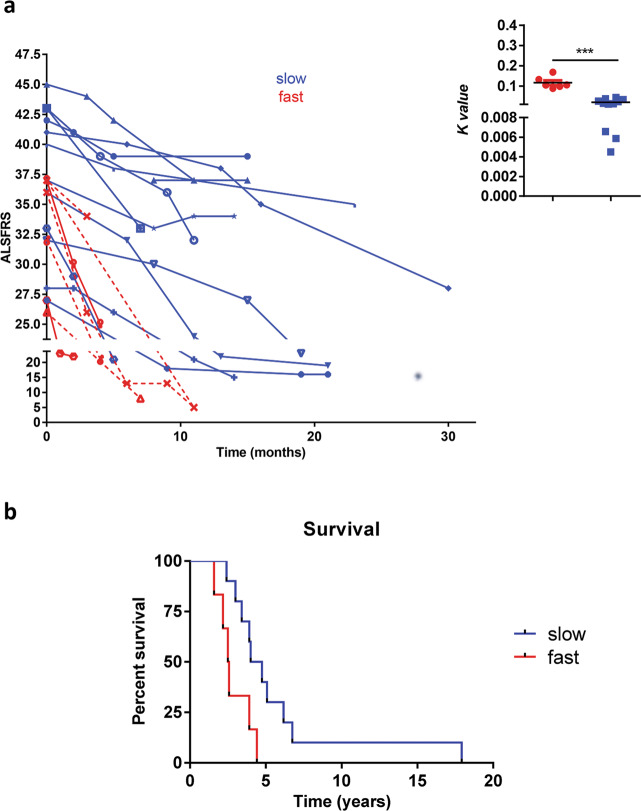
ALS-FR score decline and patient survival during the whole study. **a** Exponential regression model for the ALS-FRS score during the study. Inset: *K* value decline parameters of fast progressive patients were significantly higher than slow progressive patients (Mann–Whitney test ***p = 0,0002). Lifelong duration **b** was significantly different in slow and fast progressive patients (Log-rank Mantel–Cox test *p* = 0.0256) *N* = 11 (slow), *N* = 6 (fast).

We initially associated the ALS-FR score, collected during the whole study, to an exponential model (Fig. 2a)^17^ to obtain a rate decline value (*K value*) for each patient; successively, we categorized the ALS-FR score into two groups using a k-means cluster categorization of the *K value* decline parameters^18^. Thus, the patient’s cohort was divided into two significantly different sub-cohorts (inset in Fig. 2): 11 patients with lower rate decline—the slow progressive patients (*K value* 0,02024 ± 0,01392)—and 6 with higher rate decline—the fast-progressive patients (*K value* 0,1167 ± 0,02923); the categorization was further corroborated by survival curves analysis (Fig. 2b).

To understand whether miRNAs modulation was related to stage disease, we analyzed the expression levels of two myo-miR (miR-206 and miR-133a) together with the three of the newly identified deregulated miRNAs (miR-199a-5p, miR-151a-5p, and miR-423–3p) in the cohort of patients stratified for disease stage. Indeed, we divided the patient cohort in 4 stages classified as mild (I), moderate (II), severe (III), and terminal (IV) (Table 1) on the basis of health state of patient (HSS) estimated by ability to speak and to perform physical functions in carrying out activities of daily living^19,20^.

**Fig. 3:**
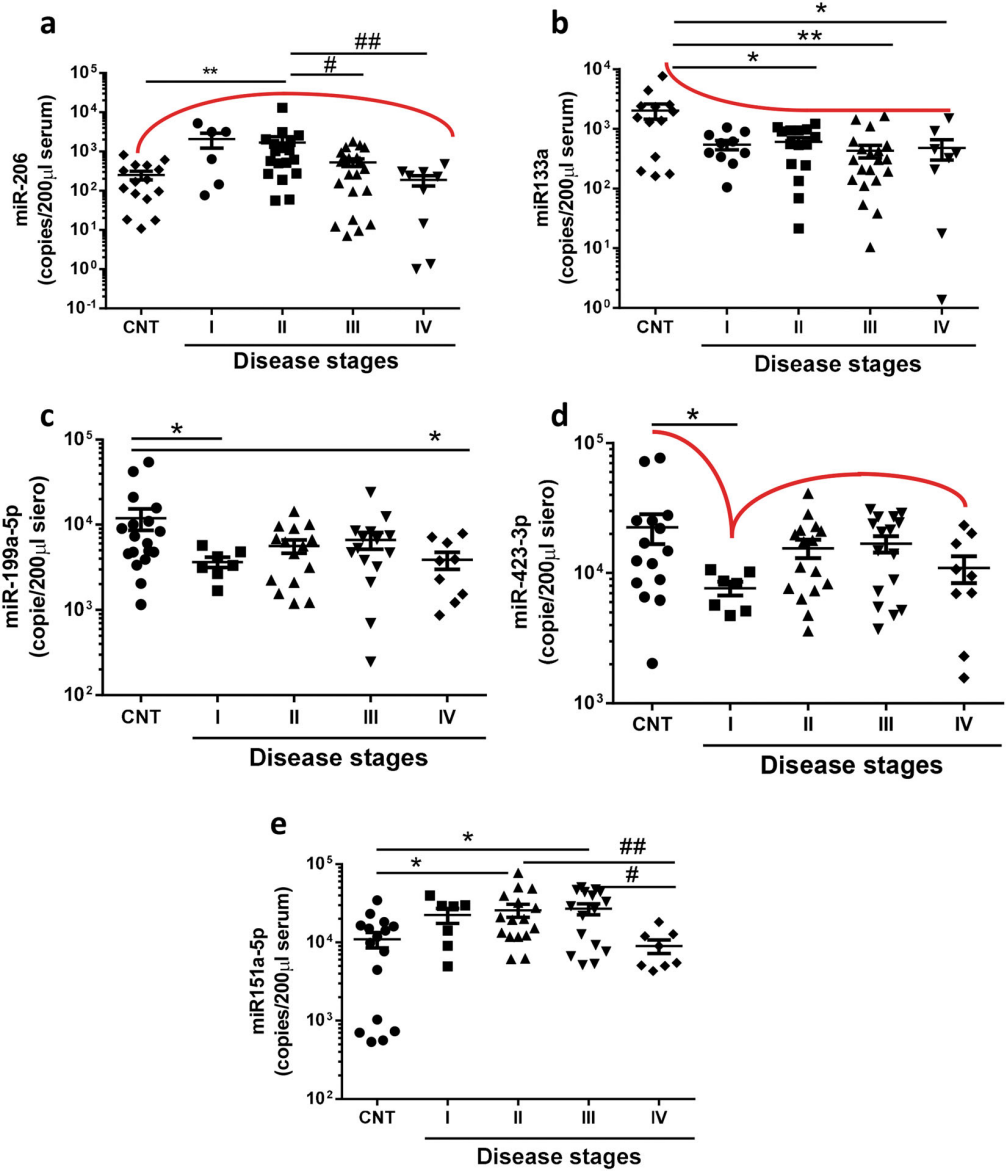
MiRNAs expression level at different stages of the disease. MiRNAs absolute RT quantification indicted that **a** miR-206, **b** miR-133a, **c** miR-199a-5p, **d** miR-423-3p and **e** miR-151a-5p are differentially modulated in ALS patients at mild (I) moderate (II), severe (III), and terminal (IV) stages compared to control individuals. Graphs indicate mean ± SEM; **a** *N* = 15 (CNT), 6 (I), 18 (II), 21 (III), 9 (IV); **b** *N* = 13 (CNT), 10 (I), 15 (II), 20 (III), 8 (IV) **c** *N* = 18 (CNT), 7 (I), 15 (II), 15 (III), 9 (IV); **d** *N* = 15 (CNT), 7 (I), 16 (II), 16 (III), 9 (IV); **e** *N* = 16 (CNT), 7 (I), 16 (II), 16 (III), 8 (IV); Mann–Whitney test: control vs stages *p < 0.05 **p < 0.01; Mann–Whitney test between stages ^#^*p* < 0.05 ^##^*p* < 0.01.

As shown in Fig. 3a, we observed that circulating levels of miR-206 were significantly increased in the moderate stage of the disease (healthy control 249.9 ± 62.7 copies, moderate 1686 ± 691.5 copies) and decreased of about 74.51% (527.3 ± 117.0 copies) and 90.96 % (186.9 ± 55.59 copies), respectively in the severe and terminal stage if compared to the mild one (Fig. 3a).

At the same time, miR-133a and miR-199a-5p levels were down-modulated all along disease progression. In particular, miR-133a significantly decreased in moderate, severe, and terminal stages of the disease (respectively −70.0%, −78.8%, −76.4%), whereas miR-199a-5p remained significantly lower in both the first and in terminal stage of the pathology (miR-133a control 2033 ± 579.9 copies, moderate 611.7 ± 102.0 copies, severe 430.1 ± 101.6 copies, terminal 479.6 ± 179.7 miR-199a-5p control 11944 ± 3376 mild 3646 ± 516.9 terminal 3863 ± 882.0 copies) (Fig. 3b, c).

Of note, miR-199a-5p and miR-423–3p were the only two miRNAs that resulted down-modulated at the first stage if compared to healthy control individuals (miR-423–3p control 22525 ± 5820 copies, mild 7630 ± 917.2 copies) (Fig. 3c, d). Interestingly miR-151a-5p remained at higher levels compared to those of healthy control individuals during disease progression with just a significant drop of its levels at the terminal stage of the pathology similarly to all the analyzed miRNAs (miR-151a-5p control 10943 ± 2435 copies, moderate 25703 ± 4856 copies, severe 26917 ± 4302 copies, terminal 8995 ± 1771copies) (Fig. 3e).

All together these data demonstrate that miR-206, miR-133a, miR-151a-5p, miR-199a-5p and miR-423–3p are modulated during disease progression and they can be considered good biomarkers of most ALS disease stages.

### MiRNAs expression levels are differentially modulated in slow and fast disease progressing patients

**Fig. 4:**
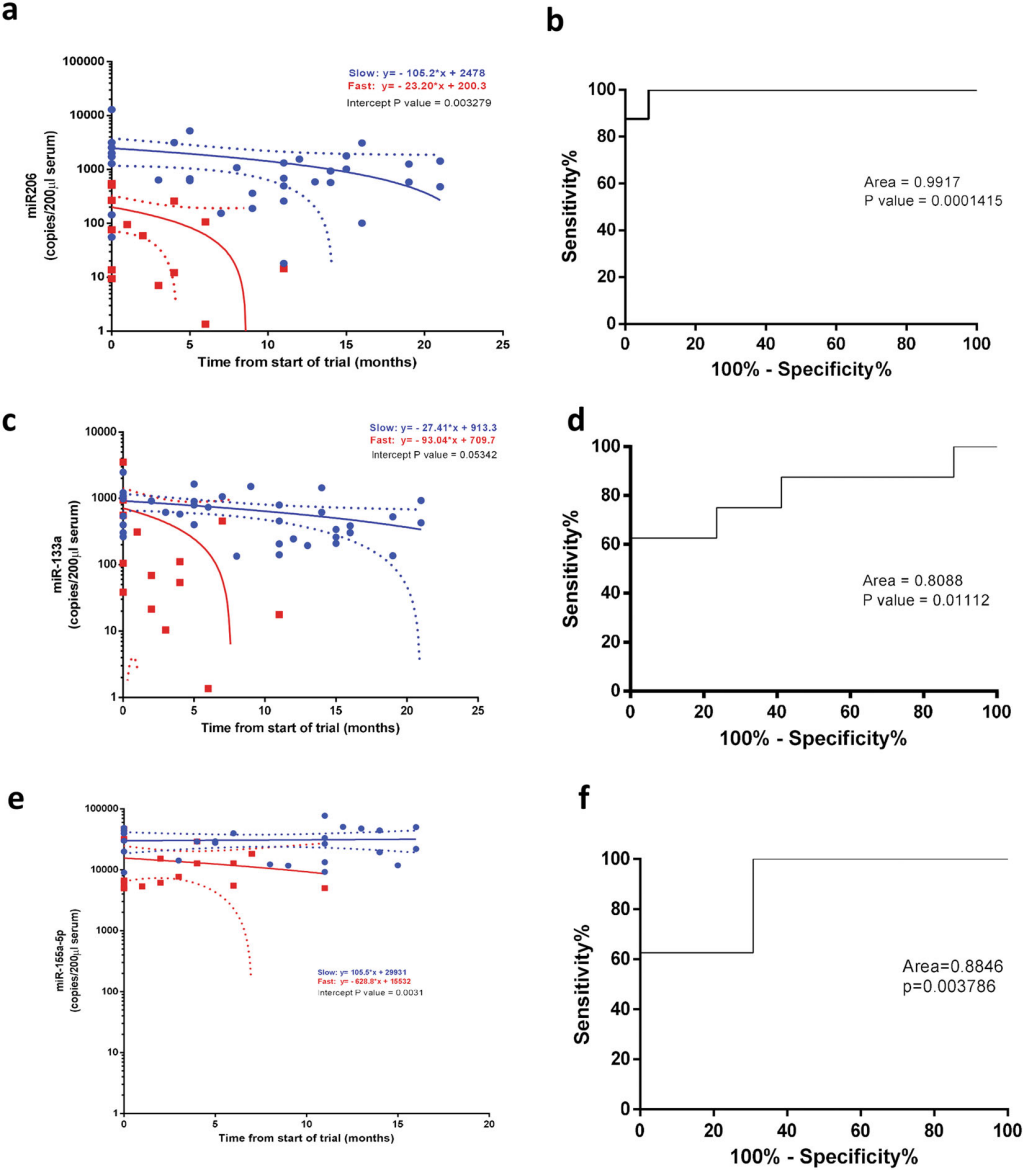
MiRNA expression levels in slow and fast disease progressing patients. Linear regression analysis of **a** miR-206, **c** miR-133a, **e** miR-151a-5p copies number in fast (red line) and slow (blue line) progressing patients. Roc curve analysis of **b** miR-206 **d** miR-133a, **f** miR-151a-5p copies number in fast and slow progressing patients at time of enrollment. *N* = 11 (slow), *N* = 6 (fast).

To define potential different distribution of microRNAs in patients with different progression of the disease, we plotted the copies number of miR-206, miR-133a, miR-151a-5p for each patient divided in fast and slow progressive groups, from enrollment to the clinical end point (Fig. 4a, c, e).

Interestingly, most of the slow progressive patients showed higher level of the miRNAs analyzed at the time of enrollment, while fast progressive disease patients showed lower copies number of miRNAs during disease progression. (Fig. 4a, c, e).

In particular, the linear regression analysis of miRNAs copies number during disease progression highlighted a significant difference between the intercepts of fast and slow progressive linear regression curves for miR-206, miR-133a, miR-151a-5p (Fig. 4a, c, e), whereas linear regression analysis of miR199a-5p and miR423–3p revealed no statistical significance for these miRNAs (Supplementary Fig. S2a and S2b).

Roc analysis of the miRNAs levels, performed at time of enrollment, revealed that the copies number of miRNAs can discriminate fast and slow progressing patients at early stage of the disease (best cut-off miR-206 ≤ 145.1 copies/200 μl serum, miR-133a ≤ 108.2 copies/200 μl serum, miR-151a-5p ≤ 11183 copies/200 μl serum) (Fig. 4b, d, f).

Overall, these data demonstrate that higher level of miR-206, miR-133a, and miR-151a-5p can predict a slow progression of the disease and a better prognosis.

### Creatin phosphokinase, albumin and alkaline phosphatase levels are differentially modulated in slow and fast progressive patients

ALS patients show distinct serological profiles and, therefore, numerous hematological factors are usually analyzed in relation to severity of clinical status of the ALS patients^20^. Here we investigated the serum levels of creatin phosphokinase (CPK), albumin and alkaline phosphatase (ALP), which are considered good markers of individuals health as they reflect the state of muscle mass, the inflammatory and nutritional status and liver functionality of the individuals enrolled in the study^18^.

**Fig. 5:**
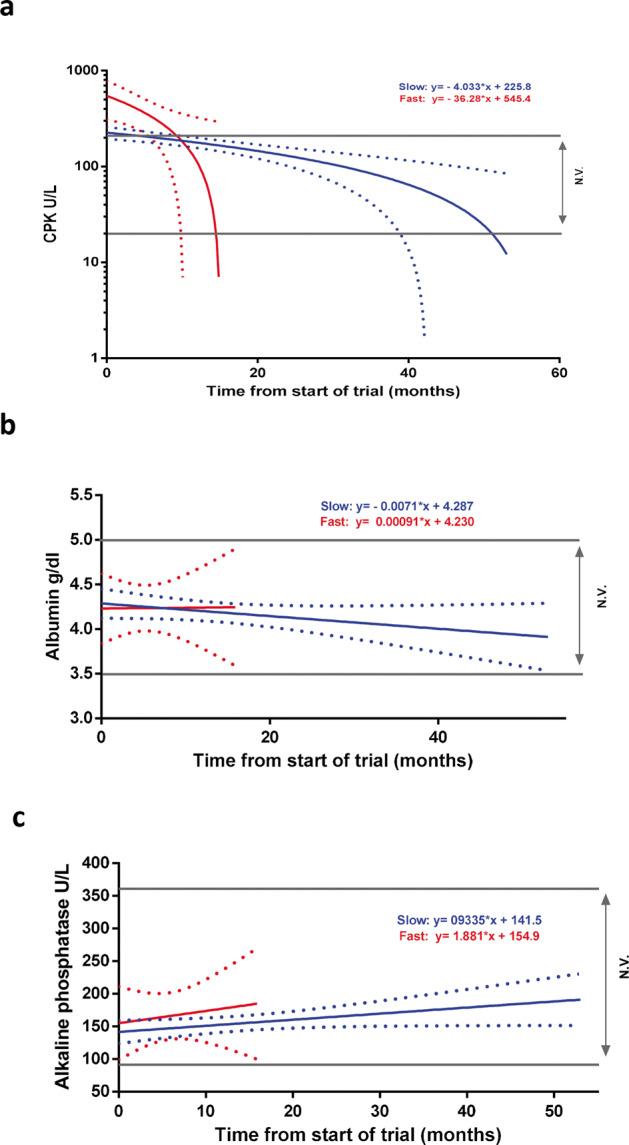
CPK levels in slow and fast progressing patients. Linear regression analysis of **a** CPK, **b** albumin and **c** alkaline phosphatase in fast and slow progressing patients. CPK levels decrease in both group with higher rate decline in the fast progressing patients compared to the slow one. Albumin and Alkaline levels do not change in both groups **a** *N* = 11 (slow), *N* = 5 (fast); **b** *N* = 10 (slow), *N* = 4 (fast); **c** *N* = 9 (slow), *N* = 3 (fast).

We analyzed serum level of CPK, Albumin and ALP during disease progression in both slow and fast progressing patients. As reported in Fig. 5a, the CPK levels decrease in both fast and slow progressing patients in line with previous evidence^21,22^, and the linear regression analysis highlighted a significant difference in the slope of the CPK linear curve. In particular, the slope or rate decline of the slow progressing patients resulted significantly smaller compared with the fast progressing ones (*p* ≤ 0.001).

In contrast, we did not observe any significant difference in the slope of the linear regression curve of Albumin and ALP. As shown in Fig. 5 the levels of Albumin and ALP remained flat or slightly increased in fast progressive patients if compared to slow one (Fig. 5b, c).

All together these data suggest that CPK hematic levels can be useful for clinical evaluation of ALS patients and represent a good support for the prognostic significance of miR-206, miR-133a and miR-151a-5p in ALS disease.

## Discussion

ALS is a multifactorial and multisystemic pathology in which severe alterations in several tissues and cell compartments, such as motor neurons, glia, and muscle, act synergistically to affect disease onset and progression^1^. Due to the complexity of the pathogenetic mechanism, the progression of the disease is highly variable proceeding in affected individuals with different aggressiveness and velocity^23^. The reason for the great variability in disease duration have not been elucidated, therefore scientific and clinical investigations in ALS field need an accurate stratification of patient cohort, according to functional decline and disease stage. This goal can be achieved by the discover and definition of molecular biomarkers that can help to perform an earlier and more accurate diagnosis with the opportunity to start an earlier treatment able to modify the disease course, to efficiently enroll patients in clinical trials, and to monitor the disease progression and identify patients who will respond better to a specific therapeutic approach.

Over the last two decades, intensive work has been carried out to find consistent biomarkers for ALS but, unfortunately, none of these biomarkers has been currently translated into a practical diagnostic tool^24^.

In our study, we examined the absolute levels of serum circulating miRNAs in ALS patients divided in four groups—mild, moderate, severe and terminal stages—according to the ALS-FRS, a parameter that define the clinical score of the pathology.

We initially focused our analysis on miRNAs that resulted differentially modulated in ALS serum subjected to NGS analysis. Three down-regulated miRNAs were identified and validated by qPCR (miR-151a-5p, miR-199a-5p, miR-423–3p) and where analyzed in detail. Few miRNAs, previously identified from other research groups up-regulated in ALS patients (e.g. miR-338–3p, miR-206 and miR-133^25–28^), were not detected by the analysis of the sequencing data probably because in terms of absolute quantities they are less expressed and therefore more difficult to be detected through NGS. Among them, we decided to analyze the expression of two important myomiR such as miR-206 and miR-133a. Although their de-regulation in ALS patients was already known^25,26^, no one had previously correlated their expression with different stages and progression of the pathology. Moreover, the copy number analyses confirmed that miR-206 and miR-133a are less represented if compared to the other miRNAs identified in sera, confirming the higher sensitivity of the single qPCR assays in comparison to NGS. Therefore, while the other identified miRs (miR-199a-5p and miR-423–3p) could be useful for diagnosis (they are highly expressed and consequently easily detectable), miR-206 and miR-133a seem to have, together with miR-151a-5p, a good prognostic value. We observed that low levels of miR-199a-5p and miR-423-3p are hallmarks of early phases of ALS pathology, while the levels of miR-206, miR-133a, and miR-155a-5p can distinguish ALS patients from control individuals in the mild, moderate and severe phases. Since ALS patients exhibit different velocity of disease progression, through a mathematical model that account for individuals functional decline, we were able to categorize patients of our study in fast and slow progressing individuals and we analyzed miRNAs levels during disease progression. By this approach, we provided evidence that serum levels of miR-206, miR-133a and miR-151a-5p can predict disease course and severity. We showed that high levels of expression of those miRNAs, in early stages of the ALS pathology, correlated with a better prognosis and slower progression of the disease.

Interestingly, the miRNAs analyzed, with the only exception of miR-423, have been associated to ALS disease. Notably circulating levels of miR-151a-5p and miR-199a-5p have been correlated with clinical variation of ALS parameters^29^ and the modulation of muscle-specific miRNAs such as miR-206 and mir-133a have been deeply analyzed in muscle biopsies of ALS patients, confirming their crucial role in the maintenance of muscle trophism and neuromuscular junction plasticity^26,30,31^. Indeed, Bruneteau et al.^32^. elucidated the direct correlation between muscle reinnervation mediated by miR-206 and ALS disease progression, suggesting that miR-206 expression might attenuate ALS progression, promoting the regeneration of neuromuscular junction.

Mir-199a has been described dyseregulated during microglial hyper-activation and persistent neuroinflammation, two abnormal conditions that support pathogenensis of ALS. In addition miR-151a-5p is thought to participate in the maintenance of cell viability in response to oxidative stress, its expression levels increased in the spinal cord of ALS patients, and it has been used as a non-muscle therapeutic target in ALS SOD1^G93A^ mouse models^13,29^. Interestingly, we observed the up-regulation of miR-151a-5p in the early phases of the disease and its significant down-modulation in the latest one, suggesting its involvement in the initial cell attempts to counteract disease onset and progression.

The novelty of our study stands in three main points that are crucial for therapeutic translation of our results.

Our study is the first to perform an absolute quantification of circulating miRNAs in ALS patients and therefore provides a cut off threshold to discriminate ALS slow progressive patients form fast progressive ones. Moreover, our work is the first to perform a longitudinal study that evaluates miRNAs levels during the progression of the disease of each patient, conferring prognostic significance to serum levels of three miRNAs analyzed, namely miR-206, miR-133a, and miR-151a-5p. With our study we overcome the limitation related to the absence of an ALS biomarker that can predict disease progression in relation to pharmacological treatment. In this context, the use of miRNAs levels to stratify patient’s cohort in different sub-groups depending on aggressiveness and velocity of the disease will help clinical practice to verify the efficacy of a treatment in clinical trial. Indeed, the possibility to identify, prior to treatment administration, those patients who will respond or not to a given therapy, will avoid unnecessary treatment and could allow the development of tailored therapy for each patient.

Until now, in the ALS field, miRNAs biomarkers have just been proposed to discriminate normal from pathological conditions; our study provides new putative biomarkers for diagnosis, stage prediction and prognosis for ALS disease and we believe that our results will be useful for the development of clinical assay that can serve as companion diagnostics in drug discovery advancement.

## Materials and methods

### Patients

Subjects were recruited from San Camillo Hospital—Forlanini of Rome, Policlinico Umberto I of Rome, Azienda Ospedaliera Universitaria ‘Città della Salute e della Scienza’ of Turin, Fondazione Policlinico Universitario A. Gemelli IRCCS of Rome. Patients’ characteristics are reported in Table 1. The study was approved by Ethical committee of Sapienza University of Rome Prot n. 939/17 Rif. CE 4636 and by Ethical committee of Azienda sanitaria ospedaliera “San Giovanni Battista” of Turin Prot n. 46 (18/02/2005). The whole study was performed in accordance with the Declaration of Helsinki, and all the patients provided written informed consent before the inclusion in the study.

Blood samples were collected into BD Vacutainer® SST II Advance for serum analysis, after sedimentation (1 h RT), serum was isolated by centrifugation (1500 rcf/min for 10 min, RT) and stored at −80 °C until use.

### Small RNA sequencing

Prior to RNA extraction, 1 fmole of two Mimic RNA Spike-in (cel-miR-2 UAUCACAGCCAGCUUUGAUGUGC and cel-lin-4 UCCCUGAGACCUCAAGUGUGA from *Caenorhabditis elegans*) was added each 200 µl of serum. Small RNAs were extracted by miRNeasy (Qiagen), following the manufacturer specifications for liquid samples. Small RNA libraries were generated from total RNA using TruSeq Small RNA Library Preparation Kit. Sequencing was performed on an Illumina HiSeq 2500 Sequencing system at the Institute of Applied Genomics (IGA; Udine, Italy). An average of about 9 million 50 nucleotide long single-end reads were produced for each sample. Read numbers and mapping statistics are reported in Supplementary Table S1.

Trimmomatic software^33^ was used to remove adapter sequences, setting the simple clip threshold to 8. Reads whose length after trimming was less than 18 nucleotides were discarded. Bowtie^34^ with parameters *-v 0 --best --norc -m 1* was then used to align remaining reads to microRNA spike-in sequences. Reads mapping to both exogenous miRNAs were counted using a custom Python script. Since very few reads mapped on cel-miR-2, which was absent from most of the samples, we decided to use only cel-lin-4 for normalization. We then used Bowtie with *--best* -*v 0* switches to align reads not mapping to spike-in sequences to a sequence database composed of canonical microRNAs and their putative isoforms; this database, derived from mirBase 21^35^, is available at http://cru.genomics.iit.it/Isomirage/. IsomiRage software^36^ was used to count reads mapping on the template isoforms of each miRNA. Read counts were supplied to DESeq2 R package^37^ for differential miRNA expression analysis; size factors were estimated using cel-lin-4 read counts. Scatter plot of log2 fold changes versus the mean of DESeq2 normalized counts was drawn using DESeq2 *plotMA* function. Heatmap of the sample-to-sample distance was computed after applying a regularized log transformation to count data using DESeq2 *rlog* function. To draw the expression heatmap of selected miRNAs, DESeq2 normalized counts were mean-centered and log2-transformed. The Small RNA-Seq data that support the findings of this study are openly available in the GEO database at https://www.ncbi.nlm.nih.gov/geo/query/acc.cgi?acc=GSE148097, accession number GSE148097. The code is available under request.

### RNA extraction and quantitative real-time PCR

Circulating microRNAs were extracted from 200 µl of serum by miRCURY^TM^ RNA Isolation Kit-Biofluids (Exiqon Cat #EX300112). During the extraction 1 µl of Spike-In Template Mixture (UniSp2: 2 fmoli/μl; UniSp4: 0,02 fmoli/μl; UniSp5: 0,0002 fmoli/μl) (Exiqon Cat # 203203) was added to each sample in order to allow the absolute quantification of microRNAs.

Subsequently 4 µl of the isolated microRNAs were retrotranscribed by miRCURY LNA^TM^ Universal RT microRNA PCR, Polyadenylation, and cDNA synthesis kit II (Exiqon Cat #EX339340) and cDNA was diluted 1:50.

Finally, 4 µl of cDNA were used for quantitative real time PCR through ViiA^TM^ 7 Real-Time PCR System (Applied Biosystems), using ExiLENT SYBR^R^ Green master mix (Exiqon Cat #203421) and Exiqon microRNAs specific primers.

In order to obtain an absolute quantification of the analyzed microRNAs, for each real time PCR assay a standard curve was generated, based on Unisp2 Spike-In template. cDNA of Unisp2 was diluted (1:10) to generate a standard curve of defined Unisp2 copies number.

### Quality analysis of total RNA extracted from plasma

In order to assess the robustness of the RNA isolation process and the quality of the isolated microRNAs we performed a quality control analysis.

We extracted RNA from 200 µl of serum of a random subgroup of 16 samples. Firstly, we checked for differences in the isolation yields performing qPCR analysis for RNA spike-in added at known concentration of RNA prior to RNA isolation. We observed that the purification process was efficient for all miRNAs even those expressed at low concentration (2–0.0002 fmoles/μl).

Secondly, we monitored cDNA synthesis by qPCR for spike-ins miR-cel-39–3p and Unisp6, added to the purified miRNAs just before retro-transcription.

Thirdly, we performed qPCR analysis (Exiqon Cat # 203845) for the detection of 4 microRNAs to evaluate the biological samples with regards to downstream processing. These miRNAs are miR-103 and miR-191 which are well expressed in most tissues, and miR-451 and miR-23a which are found in plasma and serum and serve as a haemolysis marker and as an internal control, respectively.

This set of assays indicated a good yield of RNA isolation and that the biological samples are of similar quality with regards to miRNAs relative concentration and haemolysis.

### Absolute quantification

For all samples analyzed the mean values of the Ct (cycle threshold) replicates of each microRNA, including UniSp2, were calculated, and the absolute microRNA copies number was extrapolated by comparison with the UniSp2 standard curve. Moreover, we applied a mathematical algorithm that considered the efficiency of extraction, retro-transcription and amplification procedures of UniSp2 and selected miRNAs, to overcome experimental bias and calculate the real copy number of circulating microRNAs in the 200 µl serum analyzed.

### Statistical analysis

Statistical parameters including sample sizes (*n* = number of subjects per group), the statistical test used, the statistical significance is reported in Figure legends. Unless otherwise indicated, *p*-values for simple pair-wise comparisons were performed using a two-tailed unpaired and nonparametric Mann–Whitney *U* test (*p < 0.05, **p < 0.005, ***p < 0.0005) and graph values are reported as mean ± SEM (error bars). To determine the minimum number of subjects for adequate study power (power > 0.8) the ClinicCalc sample size calculator was used. Statistical analysis, including ROC curve analysis was performed using GraphPad PRISM 6 software and best cut-off was by Youden’s index calculation.

### Serological profile analysis

Blood was collected and centrifuged at 12,000 rpm for 10 min. Serum was separated and stored at −80 °C. Creatin phosphokinase (CPK), albumin and alkaline phosphatase (ALP) determination was performed by standard spectrophotometric analysis by using Atellica CH CK_L, Atellica CH Alb, Atellica CH ALP_2c kits and analyzed by the Atellica CH Analyzer system. Absorption at 340/596 nm. 596/694 nm, 410/478 nm was measured respectively for CPK, Albumin, ALP according to manufacturer instructions.

## Supplementary information

Supplementary Figure Legends

Supplementary Table S1

Supplementary Figure S1

Supplementary Figure S2.

## Data Availability

The Small RNA-Seq data that support the findings of this study are openly available in the GEO database accession number GSE148097.
