# Supplementary material for: A longitudinal study defined circulating microRNAs as reliable biomarkers for disease prognosis and progression in ALS human patients
Source: Cell Death Discov. 2021 Jan 11;7:4. doi: 10.1038/s41420-020-00397-6 (PMC7801652; doi:10.1038/s41420-020-00397-6)
Supplement: Supplementary file 1 — Supplementary Figure Legends [file 41420_2020_397_MOESM1_ESM.docx]

**Supplementary Figure Legends**

**Supplementary Table S1** Read numbers and mapping statistics. Cnt: healthy control subjects; als: ALS patients.

**Supplementary Fig. S1** (a) Demographic characteristics of the cohort of patients and healthy controls included in the NGS analysis. (b) Heatmap of the sample-to-sample distances built on the rlog-transformed miRNA expression values obtained from Small RNA Sequencing experiment. The dendrograms describe the hierarchical clustering of the samples based on sample distances. (c) Demographic characteristics of the cohort of patients and healthy controls included in the validation by qPCR.

**Supplementary Fig. S2** Linear regression analysis of miR-199a-5p (a) and miR-423-3p (b) copies number in fast (red line) and slow (blue line) progressing patients.
