# Supplementary material for: A longitudinal study defined circulating microRNAs as reliable biomarkers for disease prognosis and progression in ALS human patients
Source: Cell Death Discov. 2021 Jan 11;7:4. doi: 10.1038/s41420-020-00397-6 (PMC7801652; doi:10.1038/s41420-020-00397-6)
Supplement: Supplementary file 2 — Supplementary Table S1 [file 41420_2020_397_MOESM2_ESM.docx]

| **Sample Name** | **Raw reads** | **Reads after preprocessing** | **Reads mapped to isoMiRS** | **Reads mapping to template miRNAs** |
| --- | --- | --- | --- | --- |
| cnt1 | 12690042 | 7604388 | 1814561 | 1782701 |
| cnt2 | 4376956 | 2490977 | 281491 | 270212 |
| cnt3 | 9731430 | 4862155 | 756240 | 740841 |
| cnt4 | 11920150 | 7604023 | 1754401 | 1707686 |
| cnt5 | 11610143 | 6466109 | 1381408 | 1349862 |
| cnt6 | 10765442 | 5360848 | 528393 | 503547 |
| als2 | 8089221 | 3593832 | 260901 | 258708 |
| als3 | 8696341 | 3761751 | 305772 | 301744 |
| als4 | 9935865 | 4152927 | 293169 | 289467 |
| als6 | 7429152 | 3105975 | 192230 | 187073 |
| als8 | 9579962 | 4218212 | 306838 | 302312 |
| als9 | 5076417 | 2749699 | 326976 | 319114 |
| als10 | 4370484 | 1704892 | 155873 | 153292 |
| als15 | 6431705 | 3300429 | 289189 | 281203 |
| als16 | 7960647 | 3495434 | 232544 | 226809 |
| als21 | 10929302 | 5997971 | 1044555 | 1021246 |
| als22 | 11922806 | 6705831 | 1309993 | 1285364 |
| als23 | 11997562 | 7698150 | 2209494 | 2179207 |
| als24 | 10858359 | 6705010 | 1600220 | 1587441 |

**Supplementary Table S1**
