# Supplementary figures and images for: A longitudinal study defined circulating microRNAs as reliable biomarkers for disease prognosis and progression in ALS human patients
Source: Cell Death Discov. 2021 Jan 11;7:4. doi: 10.1038/s41420-020-00397-6 (PMC7801652; doi:10.1038/s41420-020-00397-6)

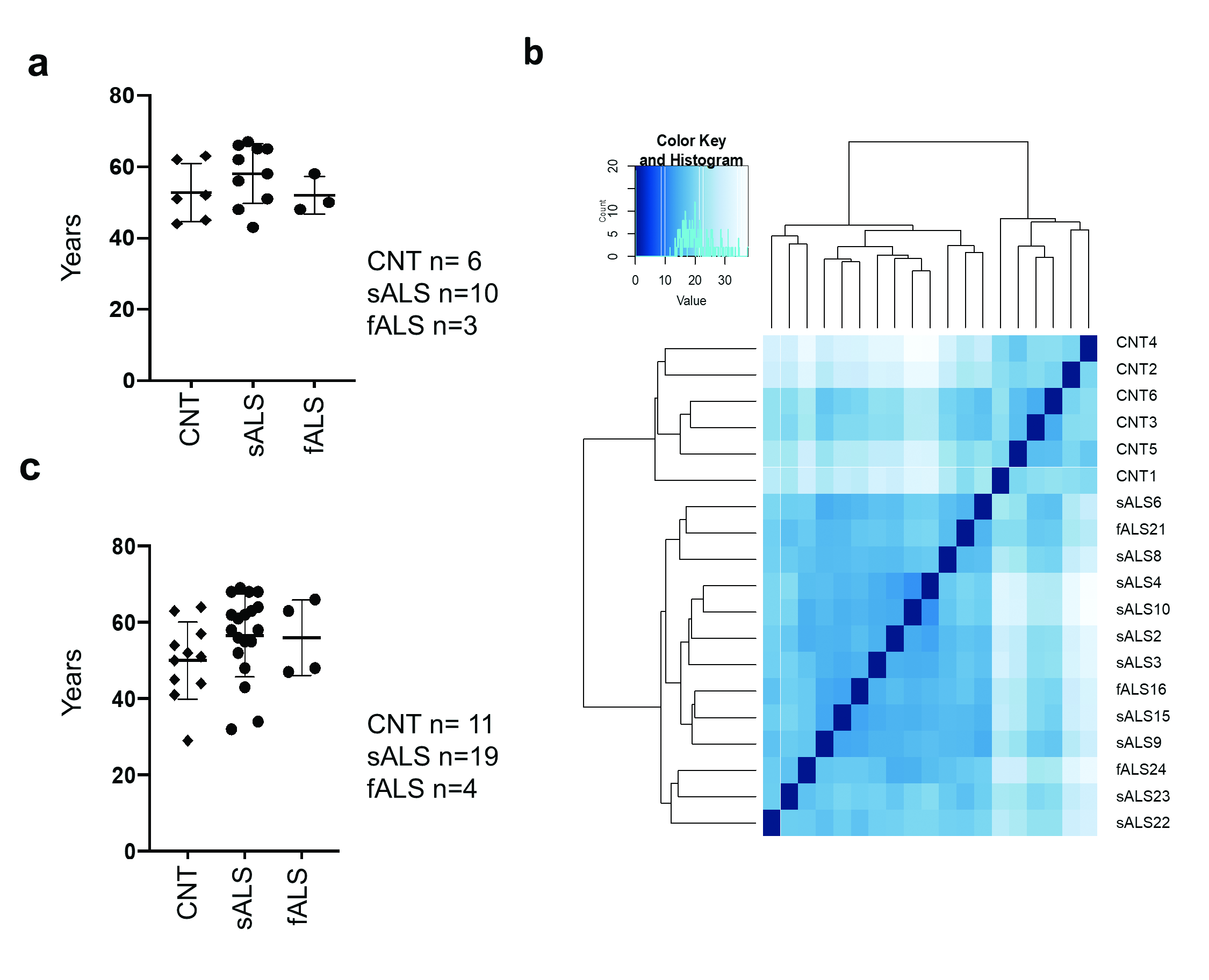

Supplement: Supplementary file 3 — Supplementary Figure S1 [file 41420_2020_397_MOESM3_ESM.tif]

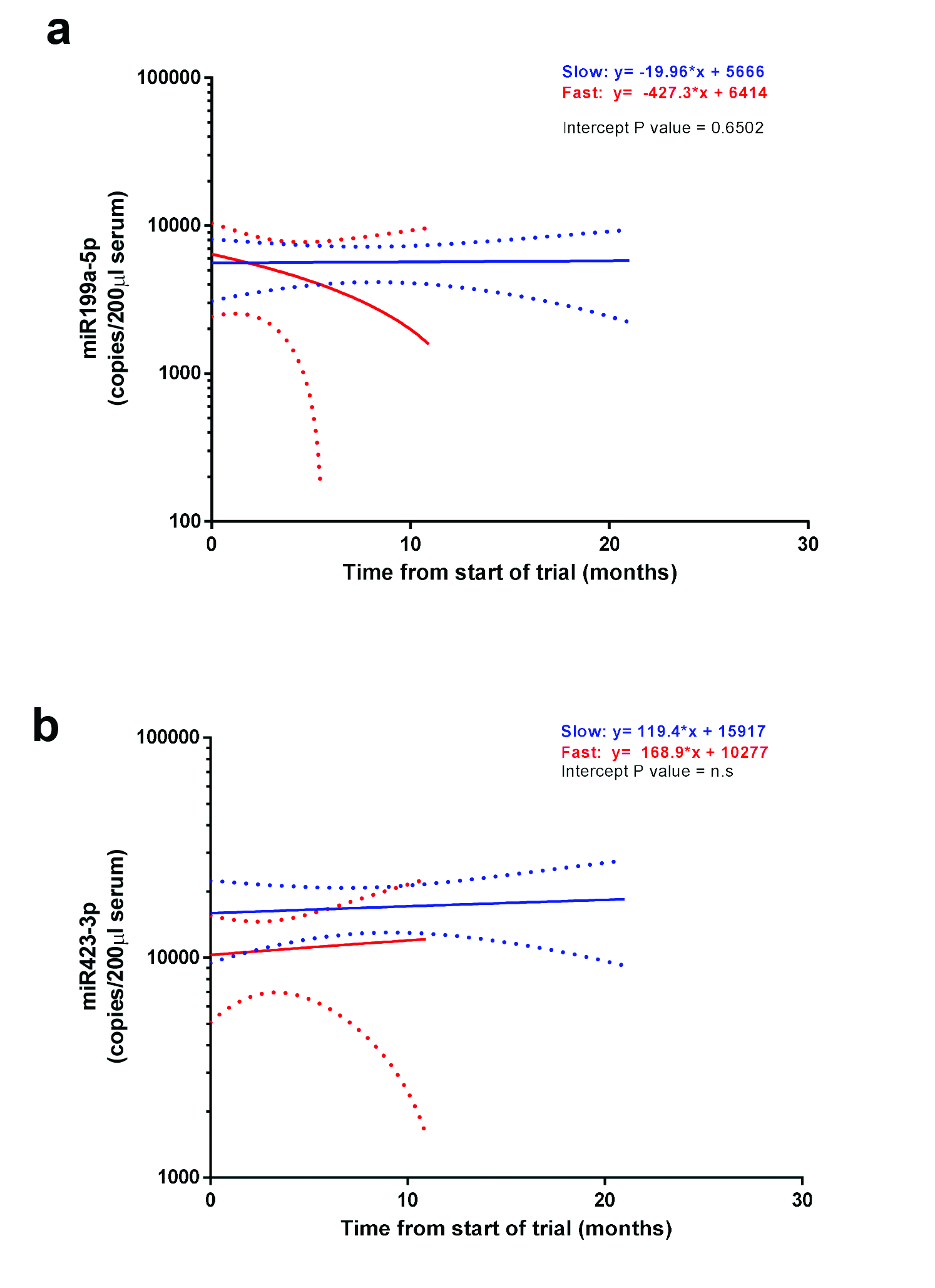

Supplement: Supplementary file 4 — Supplementary Figure S2. [file 41420_2020_397_MOESM4_ESM.tif]
